# PM2.5 Pollutant Concentrations in Greenspaces of Nanjing Are High but Can Be Lowered with Environmental Planning

**DOI:** 10.3390/ijerph18189705

**Published:** 2021-09-15

**Authors:** Binghui Yang, Ye Chen

**Affiliations:** Digital Landscape Architecture Lab of Southeast University, Landscape Architecture Department, School of Architecture, Southeast University, Nanjing 210096, China; 220200230@seu.edu.cn

**Keywords:** urban greenspaces, PM2.5, spatial-temporal distribution, influencing factors, the elderly

## Abstract

Small-scale greenspaces in high-density central urban districts serve as important outdoor activity spaces for the surrounding residents, especially the elderly. This study selects six small-scale, popular greenspaces with distinct characteristics that are jointly situated along the same main urban artery in a high-density central urban district. Field investigations and questionnaires are conducted and combined with statistical analyses, to explore the spatial-temporal distribution and influencing factors of PM2.5 concentrations in these greenspaces. The study finds that the air quality conditions in the sites are non-ideal, and this has potential negative impacts on the health of the elderly visitors. Moreover, the difference values of PM2.5 concentrations’ spatial-temporal distributions are significantly affected by vehicle-related emissions, which have significant temporal characteristics. PM2.5 concentration is strongly correlated with percentage of green coverage (*R* = 0.82, *p* < 0.05), degree of airflow (*R* = −0.83, *p* < 0.05), humidity and comfort level (*R* = 0.54, *p* < 0.01 and *R* = −0.40, *p* < 0.01 respectively). Meanwhile, the sites’ “sky view factor” is strongly correlated with degree of airflow (*R* = 0.82, *p* < 0.05), and the comfort level plays an indirect role in the process of PM2.5 affecting crowd activities. Based on this analysis, an optimal set of index ranges for greenspace elements which are correlated with the best reduction in PM2.5 concentrations is derived. As such, this research reveals the technical methods to best reduce their concentrations and provides a basis and reference for improving the quality of small-scale greenspaces in high-density urban districts for the benefit of healthy aging.

## 1. Introduction

Rapid urbanization tends to lead to excessive levels of suspended particulate matter (PMs) in the urban ambient air, along with frequent smog and haze. Long-term exposure to air pollution not only poses a great threat to humans’ health but also fauna that occur in cities, such as birds [1,2]. In addition to observations, an increasing number of state-of-the-art models are used for the description and evaluation of PMs such as the RAMS-CMAQ model and the coupling chemistry-meteorological model [3,4,5].

Urban greenspaces are an integral part of green infrastructure. As a primary function, they provide residents with spaces for outdoor activities, which is closely associated with general health and well-being [6,7]. Meanwhile, they also play beneficial roles in improving urban air quality, alleviating urban heat islands and several other health and environmental aspects [8,9,10,11]. Studies have shown that exposure to greenspaces benefits a person’s respiratory and mental health [12,13,14,15,16], while air pollution, social interactions, physical activities and other factors have an indirect role between greenspaces and their surrounding residents’ health and well-being [17,18]. However, as the greenspaces in central urban districts tend to border urban arteries and thoroughfares, and the number of motor vehicles in China grows rapidly, the potential beneficial effects of these spaces may be diminished. Against this backdrop, it has been noted that the harm caused by exposure to air pollution is particularly prominent among developed urban districts that are both population centers and emission hotspots [19].

Towards urban greenspaces, many researchers have carried out studies using varying scales to explore the spatial-temporal distributions of particulate matter, their influencing factors and mechanisms of reduction [20,21]. Compared to macro- (like global, regional, national, urban scales) and micro-scale (like microstructures of leaves) studies, there appears to be a lack of small-scale (such as specific urban parks) studies on the influence of different greenspaces’ composition elements on their pedestrian-level PM concentrations and spatial-temporal distributions, which in fact are closely associated to the health and group activities of surrounding residents. Some of these distinguishing factors include landscape features, geographical location and meteorological factors. Due to dynamic meteorological conditions and the heterogeneity of different greenspaces’ ground surface properties, distribution of emission sources, topography and other human activities, PM concentrations vary greatly in time and space between and within greenspaces [22,23,24]. The results are that the reduction effects on PM2.5 concentrations also vary widely at different greenspace locations [25,26,27]. One comparison of several small greenspaces in urban parks, schools and residential areas showed that location and time have an extremely significant influence on the PM2.5 concentrations [28]. Even within the same park, PM2.5 concentrations in small sites composed of different landscape elements vary significantly while also varying over time [29]. Other researchers have studied different small-scale sites within a comprehensive park and found that the spatial distribution of PM2.5 concentrations varied minimally [30]. Overall, existing research shows that PM2.5 concentrations have notable seasonal and daily variations, exhibiting significant differences between macro-regions; meanwhile, the research results of the effect on their spatial-temporal distributions from small-scale greenspaces which are most closely related to people’s daily lives appears non-uniform. This study seeks to better explore this non-uniformity to improve the benefits such greenspaces may provide at reducing PM2.5 concentrations.

The environmental factors affecting PM concentrations at a greenspace site include the percentage of green coverage, sky view factor (SVF), vegetation quantity, plant community structure, vegetation density, canopy density, configuration mode, total greenspace area, canopy volume coverage and plant diversity [31,32,33,34]. Urban districts with high levels of green coverage can help reduce PM concentrations, and have been shown to be typically negatively correlated with PM concentration levels [35]. Water environments within greenspaces affect PM2.5 diffusion and deposition as well, which is related to changes in airflow, temperature and humidity caused by the evaporation and cooling of water [33,36]. In addition, the impact of vegetation on PM concentration also depends on surrounding traffic density and the relative location of emission sources [37,38].

Meteorological parameters, such as rainfall, snowfall, wind speed and direction, temperature and humidity, are another important set of factors affecting PM concentration and exert varying effects on PMs of different sizes [24,39,40,41,42]. Within a certain range, PM concentration is negatively correlated with temperature [43] and positively correlated with relative humidity [44,45]. Good natural ventilation and airflow can accelerate the local diffusion of pollutants and dilute their concentration, but high wind speeds can also raise dust from the ground, causing secondary air pollution, while wind direction may affect the location and origin of pollutant [46,47]. At the same time, thicker and larger plant canopies can reduce wind speed, typically resulting in an increase in PM concentration [48]. Finally, evapotranspiration (the sum of evaporation from the land surface plus transpiration from plants) can change a site’s temperature and humidity and further affect the related PM deposition processes.

In summary, several influencing factors of urban greenspaces, including their composition and meteorological conditions, heavily influence their associated PM concentrations. These factors also cause certain differences in the spatial-temporal distributions of PM. Currently, many studies have primarily focused on PM2.5 concentrations by analyzing macro-scale regional variations or, on the micro-scale, by looking at the effects of individual plants. In contrast, few studies have focused on the linkages among meteorological factors, greenspace elements, pollution sources distribution and PM concentrations. Furthermore, there remains great uncertainty among research conclusions regarding the detailed influence of meteorological factors and landscape elements on the concentration of suspended PM.

At present, the global aging problem has drawn great deal of attention in most parts of the world [18]. In 2020, the total number of elderly people aged 60 and above in China was 264 million, accounting for 18.7% of the total population. Nanjing had 1.77 million people aged 60 and above, accounting for 19.0% of the city’s total population [49,50]. China has, accordingly, proposed a national strategy for health aging. Moreover, China’s urbanization development is inevitably leading to an increasingly serious fragmentation of urban greenspace [51]. Subsequently, fragmented small-scale greenspaces have become an important venue for outdoor activities of residents in high-density urban districts. According to official meteorological data, there are varying degrees of smog and haze among the greenspaces of Nanjing. Long-term exposure to smog and haze poses especially severe health risks to the elderly.

In light of the above discussion and literature review, and while urban land becomes increasingly limited, this paper has chosen to focus on the elderly populations who are the largest user group of greenspaces in China [52,53,54]. With a comprehensive consideration of pollution sources, urban rivers, meteorological conditions and other environmental factors, in addition to consideration of distinctive greenspace characteristics and distance from main urban arteries, this study selected several typical small popular greenspaces dispersed across high-density central urban districts in Nanjing. Sociological research methods such as evidence-based measurements, questionnaires and interviews were adopted, while relevant statistical software was used for the corresponding quantitative analysis. In studying these urban greenspaces, a quantitative and comprehensive analysis was undertaken on the spatiotemporal distribution patterns of the sites’ PM2.5. In addition, further analysis was undertaken on the relationships between environmental factors (temperature and humidity, wind speed and direction), greenspace elements (green coverage, water coverage, airflow openness, SVF) and PM2.5 concentrations. This study also explores the relationships between exposure to urban greenspaces and air pollution on the physical and mental health of the elderly, and thereby hopes to help promote healthy aging and better understanding of how to create the most suitable landscapes for the elderly, while also providing reference for the optimizing of greenspace elements.

In particular, this paper aims to study the following questions:

1. What are the spatial-temporal distributions characteristics of PM2.5 concentrations in small-scale urban greenspaces in high-density central urban districts?
2. What are the influencing factors and mechanisms of PM2.5 concentrations in small-scale greenspaces?
3. How does information on the smog and haze in urban greenspaces affect the decisions of the elderly on going out and visiting such spaces?
4. What are the relevant implications of this study for urban planning and design?

## 2. Methodology

This research focuses on the greenspaces dispersed across high-density central urban districts to explore the spatial-temporal distribution of PM2.5 and the corresponding influencing factors. Various research methods were used including evidence-based research, structured questionnaires and interviews, whose results were then combined with relevant statistical analyses (Figure S1).

### 2.1. Research Sites

**Figure 1:**
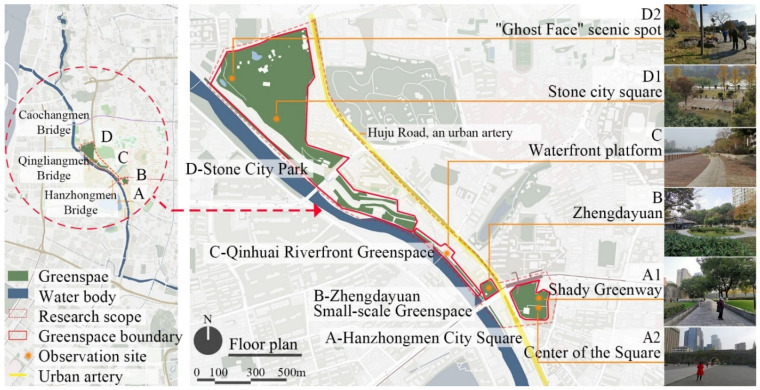
Locations of surveyed Nanjing greenspaces.

**Figure 2:**
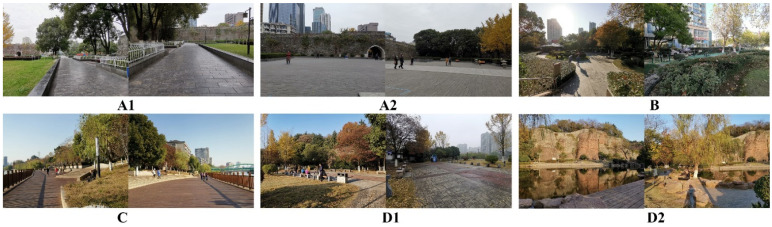
Site photos. (**A1**) Shady Greenway (**A2**) Center of the Square (**B**) Zhengdayuan (**C**)Waterfront platform (**D1**) Stone City Square (**D2**) “Ghost Face” scenic spot.

Nanjing is a central city of the Yangtze River Delta region in eastern China (31°14′~32°37′ N, 118°22′~119°14′ E). It has a subtropical humid climate. Known as the “Mother River of Nanjing”, the Qinhuai River flows through the central urban districts of the city and is the largest regional river in Nanjing. There are a large number of old residential areas along the Qinhuai River and this study’s preliminary survey found that the elderly have become the main users of the greenspaces along the Qinhuai River. Therefore, based on the above literature review and survey, this study focuses on the health and well-being of the elderly population. It should be pointed out that in China, officially, seniors are people aged 60 years or more [49]. However, female workers generally retire at the age of 50 and also become the main users of parks and urban greenspaces [54]. As such, the elderly in this study and the targeted interviewees are people aged 50 and above. In order to avoid researching a single type of greenspace which potentially has no obvious differences in PM2.5 concentration trends, this study has attempted to select a diverse set of greenspace sites by comprehensively considering differences in their physical environments, environmental preferences of the elderly and their perpendicular distance from the same shared urban artery. Subsequently, four typical urban greenspaces located between the same urban artery and the Qinhuai River were selected as research sites, including a large city square adjacent to the city street (Site A), a small-scale greenspace located between the city street and the river (Site B), an urban riverfront greenspace (Site C) and an urban park on the riverfront of the Qinhuai River (Site D). According to different distances to adjacent urban roads, Sites A1 and A2 were then selected from within Site A. Based on the linear characteristics of its riverfront park and contrasting landscapes, sites D1 and D2 were selected from within Site D. Site D1 features a hard-surface public square (D1) while Site D2 is located between the riverfront and a hill landscape of approximately 35 m in height. The six sites share the characteristic of being the sight for a highly dense and diverse set of activities which serve as popular gathering areas for the surrounding elderly communities. At the same, the sites are distinguished by differences in their greenspace elements and distance to primary pollution sources (Figure 1 and Figure 2).

Corresponding to the dual dimensions of physical and mental health, this study divides observed elderly activities within the sites into two types, physical activities (PA) and social activities (SA), although it is noted that the two categories can often overlap. Field observations of visitor activities (Table 1, Figure S2): in Sites A1 and A2, the elderly mainly engaged in social activities such as playing chess and cards, chatting and sitting idly. In Sites B and C, elderly visitors (This paper will use the term elderly visitors and visitors interchangeably, with the understanding that the greenspace site visitors are predominantly elderly visitors in China) mainly engaged in physical activities, such as playing ball games and using fitness equipment. In D1 and D2, the proportions of social and physical activities among the elderly were both high.

### 2.2. Survey Period and Times

According to the air monitoring data of the Nanjing Meteorological Bureau over the past five years (2015–2019), of the city’s four seasonal periods, Nanjing’s winter season has had the most severe smog and haze. Based on this, surveying of the sites was decided to be conducted from December 2020 to January 2021, covering both weekdays and weekends, and in consideration of including different weather conditions (sunny, cloudy, rainy and post-snow). A total of 138 datasets were collected from 23 days over this period. Based on targeting the sites’ peak periods of elderly visitors, the specific surveying period for the sites was set at 8:00–11:30 a.m. and 13:30–16:30 p.m. In order to help ensure PM2.5 values were relatively stable and consistent, the physical environment data of the six observation sites was collected in a continuous fashion within sequential half-hour periods during the surveying periods.

### 2.3. Data Collection

#### 2.3.1. Physical Environment Data Collection

A handheld aerosol monitor (TSI-8534 DUSTTRAK DRX, Shoreview, MT, USA) was used to monitor the concentration of inhalable particles at the different sites. The instrument can simultaneously measure the mass concentration of different particle sizes, including PM1, PM2.5, PM10. A handheld anemometer (16025) was used measure the average wind speed and direction. A temperature and humidity data recorder (TESTO 175H1, Lenzkirch, Germany) was used to record the temperature and humidity in real time. Finally, a GoPro Max camera (GoPro, San Mateo, CA, USA) was used to take 360° panoramic photos at a vertical distance of 1.5 m. The shadowing contour of the picture is processed on a circular plane, which can then be used to calculate a sky-view factor (SVF) which represents the degree to which trees and buildings do not obstruct direct viewing of the sky from a centralized point within the site.

#### 2.3.2. Visitor Data Collection

While collecting the sites’ environmental data, the number of visitors and types of activity at the site were observed and recorded with the time, location and other data. In addition, structured questionnaires and interviews were carried out to quantitatively explore the awareness of elderly visitors towards smog and haze, with all interviews saved as audio recordings.

### 2.4. Basic Methodology of Data Analysis

After the basic statistics of the sites’ physical environment data and visitor data were collected and organized, this study adopted statistical software SPSS (v.25) to carry out several statistical analyses on portions of the dataset, including one-way ANOVA, correlation analysis and mediating effect analysis.

## 3. Results

The study was able to obtain cumulative data of the six sites over 23 days, including 138 datasets on visitor activities and local atmospheric conditions. Then a questionnaire was issued to the elderly while measuring environmental data. Based on the air quality of the day, the distribution was selected when the haze was severe, and 200 valid questionnaires were finally screened.

### 3.1. Spatial-Temporal Distribution Characteristics of PM2.5 Concentrations

Air pollution exposure and corresponding health effects are usually assessed by using air pollution data from fixed-location urban monitoring stations. However, a large number of studies have suggested that the pollution exposure of the local populations is often significantly underestimated by such monitoring stations [55,56]. This is because the sampling of these monitoring stations by the Meteorological Bureau is typically carried out at a high altitude and at an always fixed location, and thus its measured data are likely to contrast with those of a handheld aerosol monitor held at an adult human breathing height (1.5 m). Such directly measured data are usually higher than the official meteorological data, and the difference is even greater when air quality is relatively poor. This is why sometimes people feel that “there is a difference between the actual air quality and the weather report” in daily life. Considering the actual respiratory exposure of an active crowd of outdoor visitors, it seems necessary to measure the actual spatial-temporal changes in pollutant concentrations and exposure levels.

#### 3.1.1. General Trends of PM2.5 Concentrations

According to the Technical Regulation on Ambient Air Quality Index (HJ633-2012) issued by China’s Ministry of Ecology and Environment [57], this study’s 138 datasets show that in more than 70% of the survey periods, the air quality of the six sites was moderately polluted or above (PM2.5 ≥ 115 μg/m³), suggesting that the air quality of the sites in this study is worrying and exposes visitors to serious health risks (Text S1). By averaging the PM2.5 data of the six sites, it was found that the average PM2.5 concentration of two waterfront sites (D1 and D2) in Stone City Park (the largest greenspace included in this research) ranked the highest, and that of the center of Hanzhongmen Square (A2) showed the lowest (Table 2). Interesting, this conflicts starkly with the common sense notion that “there is less smog and haze along the riverside due to its strong winds”. The difference here may be in fact caused by temperature and humidity, which will be further discussed in Section 3.2.2.

According to the 23 days of measured data, the highest PM2.5 concentration occurred on Day 9, a cloudy day with no wind and high humidity (61.8%), while the lowest value occurred on Day 10, just one day after Day 10 and also after Nanjing just experienced heavy snowfall. As Day 10 was the first day after the snowfall, the air quality remained relatively good for three to four days after Day 9. This is assumed due to the dust falling and depositing into the ground due to rain and snow. In addition, PM2.5 concentrations levels continuously fluctuated at the different research sites during the same measurement period. The PM2.5 concentration of D1 and D2 on Day 4 is notably higher than that of other sites in the same period, which is believed to be related to a significant difference in site temperature and humidity at that time, namely, the humidity of the two sites is relatively high while the wind speed is relatively low. The second time there was a big difference in PM2.5 concentrations between the sites was on Day 22, with D1 and D2 still ranking the highest, and the difference was assumed to be related partly to the different humidity and wind speeds at that time (Figure S3).

**Figure 3:**
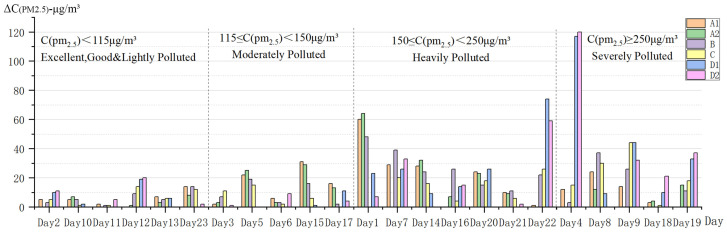
Comparison of difference values of PM2.5 concentrations.

In addition, by comparing the difference values of PM2.5 concentration of the 23 days across the six sites, it was found that when the PM2.5 concentration >150 μg/m³, that is, the average air quality reaches heavily polluted or above, the differences in PM2.5 concentration between the six sites increased significantly (Figure 3). Thus, it can be inferred that when the PM2.5 concentration is ≤150 μg/m³, the differences in the sites’ spatial layouts and environmental factors likely have little impact on their PM2.5 concentrations. The difference value within groups is calculated by the following formula, that is, the difference value between each datum in a group and the minimum value of the group:

(1) $$F\left(C\right)=C_{real}-C_{min}$$

#### 3.1.2. Spatial Distribution Characteristics of PM2.5 Concentrations

**Figure 4:**
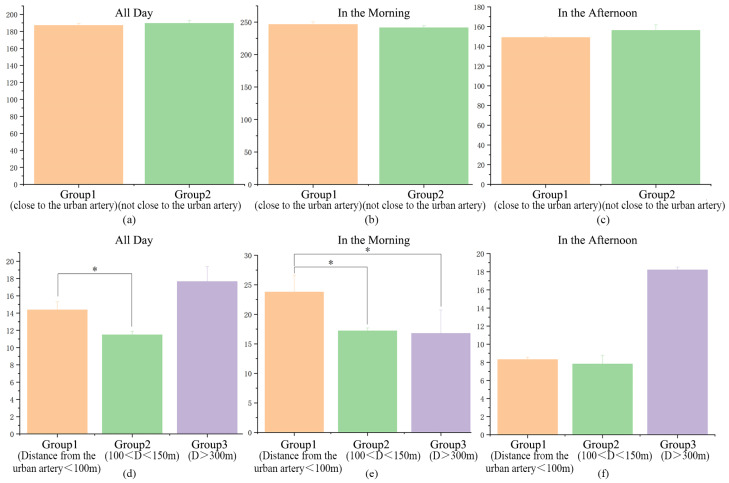
Variance analysis of the spatial distribution of PM2.5 concentrations ((**a**–**c**): PM2.5 concentration; (**d**–**f**): Difference value). * *p* < 0.05.

The six greenspace sites were grouped into two spatial location categories based on whether they were close to the urban artery or not. The results of a one-way ANOVA on these two groups and their directly monitored PM2.5 concentrations show that PM2.5 concentrations were not significantly affected by their spatial location in relation to the urban artery, regardless as to whether it was a certain period of the day or the whole day data, with differences between sites ranging from 0.53% to 2.31%. The sites were further grouped into three spatial location groups based on their shortest perpendicular distance to the urban artery, namely Group 1 (<100 m), Group 2 (100 m~150 m) and Group 3 (>300 m). The one-way ANOVA on these groups and their difference values of PM2.5 concentrations showed that there were significant differences between Group 1 and Group 2 (*p* < 0.05) when comparing their whole day data. It also showed that during the morning period, there were significant differences between Group 1 and Group 2, and between Group 1 and Group 3 (*p* < 0.05) (Figure 4).

Overall and from a macro perspective, due to the relatively close locations between the six sites (the furthest straight-line distance between the two sites is approximately 2 km), there appeared to be no great difference in PM2.5 concentrations during the same periods of time at different spatial locations. However, in terms of their difference values, the sites’ spatial locations did appear to have a significant impact on the PM2.5 concentration in greenspaces, and this was assumed to be greatly influenced by traffic pollution sources on the surrounding city roads.

**Figure 5:**
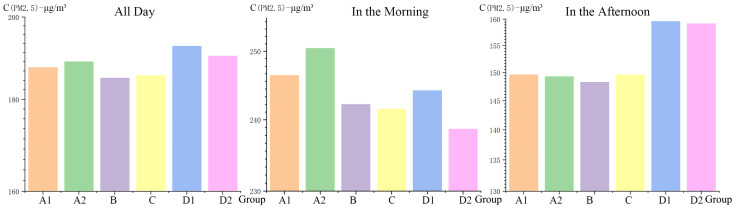
Comparison of PM2.5 concentrations across the six research sites.

As shown in Figure 5, during the morning period, Site B’s PM2.5 concentration levels are the highest because it is situated near the main urban artery and thereby is greatly influenced by rush hour traffic when large numbers of vehicles and pedestrians raise emission and dust levels. In contrast, Site D2 has the lowest PM2.5 concentration levels during this period. It can be inferred that this site is least affected by the urban traffic and pedestrian activities because it is the site furthest from the urban artery and its environment includes a hill which may further block traffic pollutants. Notably during the non-rush hour times in the afternoon period, it can be seen that PM2.5 concentrations in Sites D1 and D2 are relatively high compared with those in other sites. It is speculated that during non-peak times, Sites D1 and D2′s peak-time advantage of being relatively far from the urban artery becomes insignificant while other factors play a correspondingly larger role towards PM2.5 concentrations.

#### 3.1.3. Temporal Distribution of PM2.5 Concentration

**Figure 6:**
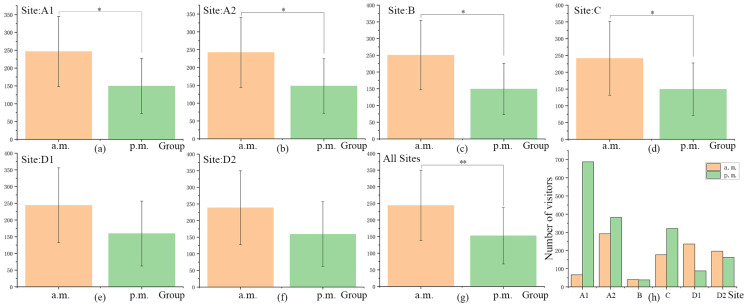
Variance analysis of temporal distribution of PM2.5 concentration and Spatial-Temporal Distribution of Visitor Activities. (**a**–**g**) Variance analysis of temporal distribution of PM2.5 concentration for each site (**h**) Spatial-Temporal Distribution of Visitor Activities. * *p* < 0.05, ** *p* < 0.01.

A one-way ANOVA was also conducted on the PM2.5 concentration data of the six sites in different time periods, and the results show that except for Sites D1 and D2 in Stone City Park, the PM2.5 concentration levels of the other four sites experienced significant differences during different time periods (*p* < 0.05). Comparing all sites shows that PM2.5 concentrations varied during different time periods (*p* < 0.001) and that PM2.5 concentrations in the morning are notably higher than that in the afternoon at all sites. This goes against the commonly held belief that the healthiest time to go out is around 9 or 10 am. Corresponding to this, the statistical results of Sites B and D’s spatial-temporal distribution of visitor activity shows the number of people in activities in the morning is greater than that in the afternoon, which indicates that there may be health risks for the elderly who are currently choosing to go out to these activities (Figure 6).

Overall, the impact of traffic pollution sources on PM2.5 concentration levels was notable. As the closest site to the urban artery, Site B had the largest difference in PM2.5 concentrations between the morning and afternoon. As explained above, this was assumed to be caused by an increased influence of traffic pollution during the morning rush hour. Conversely, Site D2’s PM2.5 concentration difference value between the morning and the afternoon was the lowest as it was the furthest site from the urban artery and had the highest proportion of water coverage. Site D2′s relatively stable PM2.5 concentration was likely largely attributable to the regulation function of its water bodies. Moreover, due to its relatively long distance from roads and relatively large proportion of water bodies, Site D2’s PM2.5 concentration in the morning was the lowest compared with all other sites, with the low impact of traffic pollution sources being particularly eye-catching.

### 3.2. Analysis of PM2.5 Concentrations Influencing Factors

#### 3.2.1. Analysis of Greenspace Elements

Small-scale greenspaces in high-density urban centers are the most frequently visited and popular sites of their surrounding residents. According to China’s Urban Greenspace Classification Standards (CJJ/T85-2017) [58] as well as the conforming to the findings of this study’s onsite field surveys, small-scale greenspaces are generally less than 1 hectare in area. In consideration of this definition and the physical characteristics of pollution sources, this study used the center points of its six dispersed research sites to delineate circular observation areas of 1 hectare within each site. Relevant indicators of greenspace elements were then observed, measured and calculated for each site based on these equally sized observation areas. Since vegetation and spatial layout are the two most important aspects affecting greenspaces, this study attempted to capture key features of these aspects by measuring and comparing the following indices across the six research sites: green coverage, water coverage, airflow and sky view factor (SVF). The collective results from the six greenspace sites show that westward and northwest winds are the dominant wind direction during the observed winter period. Additionally, considering the wind resistance characteristics of buildings and trees, as well as the semi-ventilating effects of trees, the sites’ airflow conditions were categorized into two general conditions: fully open [(S site area–Sa building area)/S site area] and semi-openness [(S site area–Sa building area–Sb tree canopy plane area)/S site area]. The 360° panorama photos were processed via Raman Pro 3.1 to calculate the SVF (Table 3).

Correlation analyses were conducted on the sites’ PM2.5 concentrations and each greenspace elements (Table 4). Irrespective of weather conditions, from the overall measurements of the 138 datasets there was a significant negative correlation found between PM2.5 concentration and degree of airflow (*R* = −0.830, *p* < 0.05). If weather conditions are controlled to be the same, that is, sunny or cloudy days are selected as the majority, and according to Beaufort Scale and on-site measurement, times where the wind speeds are greater than 0.3 m/s are further selected, the sites’ PM2.5 concentrations are significantly correlated with the percentage of green coverage and the degree of semi-open airflow (*p* < 0.05). Specifically, the higher the percentage of green coverage rate (*R* = 0.819), the higher the PM2.5 concentration, and the higher the degree of semi-open airflow (*R* = −8.887), the lower the PM2.5 concentration. In comparison, when the wind speeds are greater than 1.5 m/s, there was a significant negative correlation between PM2.5 concentrations and semi-open airflow (R = −0.833, *p* < 0.05). It is thus speculated that the effect of green coverage on PM2.5 concentrations is weakened when wind speeds exceed 1.5 m/s. A further correlation analysis of the sites’ greenspace elements revealed a positive correlation between SVF and degree of fully open airflow (*R* = 0.821, *p* < 0.05). This implies that higher SVF’s are associated with a higher degree of fully open airflow, thereby indirectly affecting PM2.5 concentrations of the site. Furthermore, there is a negative correlation between green coverage and semi-open airflow (*R* = −0.833, *p* < 0.05), while semi-open airflow is significantly positively correlated with the fully open airflow (*R* = 0.886, *p* < 0.05) (Table S1).

#### 3.2.2. Analysis of Meteorological Factors

A correlation analysis was conducted on the six sites’ PM2.5 concentrations, humidity, temperature, wind speeds and comfort levels (Table 5). Based on conventional meteorological data, from the perspective of easy accessibility and convenience of parameters, the calculation was carried out by using the Lu Dinghuang comfort formula. The formula is highly comprehensive and widely used in the field of landscape architecture, and can better reflect the comfort of the research sites [59,60,61]. The comfort level was calculated by the following formula, where *S* refers to the comfort level, *T* the temperature (°C), *RH* the relative humidity (%), and *V* the wind speed (m/s).

(2) S=0.6 (|T−24|)+0.07 (|RH−70|)+0.5 (|V−2|)

The results show that there is a strong positive correlation between PM2.5 concentrations and humidity (*R* = 0.541, *p* < 0.01). This implies the higher the humidity, the higher the PM2.5 concentration tend to be, matching the results of other researchers, such as Tian (2014) [45] and Qiu (2018) [28]. A moderately negative correlation between PM2.5 concentrations and comfort level was found (*R* = −0.400, *p* < 0.01), suggesting the higher the comfort value of a site, the lower the PM2.5 value is. In contrast, the study’s analysis found PM2.5 concentrations had a weak positive correlation with temperature and no significant correlation with wind speed. The lack of a correlation with wind speed may lie in the fact that selected sites’ wind speeds remain relatively low, with an average wind speed of less than 3.3 m/s, and thus the wind would not have a significant impact on the diffusion of PM. This result is in line with the research of Beckett (2000) [62] and Freer-Smith (2005) [63], namely that wind speed significantly affects the migration and diffusion rate of PM only within a certain range.

**Figure 7:**
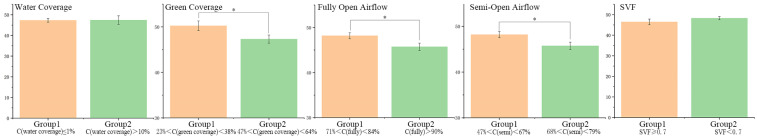
One-way analysis of variance (ANOVA) of humidity and greenspace elements. * *p* < 0.05.

On the basis of the above correlation analyses, greenspace element factors affecting the sites’ humidity were further analyzed. A one-way ANOVA was performed successively for sites’ humidity and green coverage, water coverage, semi-open airflow, fully open airflow, and SVF. The results show that the humidity is significantly correlated with the degree of green coverage and airflow openness (*p* < 0.05) but is not significantly affected by water coverage or SVF (Figure 7).

### 3.3. Correlations between Visitor Activities, PM2.5 Concentrations and Greenspace Elements

#### 3.3.1. Correlation between Visitor Activities, Meteorological Factors and PM2.5 Concentrations

A correlation analysis was undertaken on the sites’ visitor activities, PM2.5 concentrations and meteorological factors (Table S2). Importantly, it found that there is no obvious correlation between visitor activity type and PM2.5 concentration. Meanwhile, the number of visitors showed a significant positive correlation with temperature (*R* = 0.223, *p* < 0.01), and a significant negative correlation with humidity (*R* = −0.252, *p* < 0.01). These results suggest that higher site temperatures represent warmer winter weather, which could lead to larger numbers of visitors participating in outdoor activities. In contrast, higher humidity indicates wetter and colder winter weather which leads to lower numbers of such visitors. These findings can also be interpreted as representing elderly visitors’ contrastingly high sensitivity towards temperature changes and weak sensitivity towards smog and haze as they make decisions whether to participate in outdoor activities.

**Figure 8:**
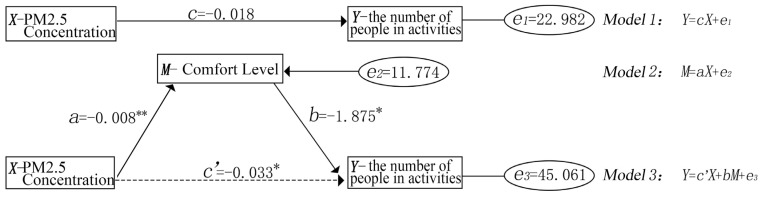
Mediating effects model of the comprehensive comfort level. * *p* < 0.05, ** *p* < 0.01.

Combined with the results in Section 3.2.2 which showed PM2.5 concentrations to be closely associated with meteorological factors, this study subsequently adopted the mediating effect analysis to further explore the role of the sites’ meteorological factors (played as mediating variable M) in the process of PM2.5 concentrations affecting visitor activities. Meanwhile, the bootstrap method was used to test the mediating effect and it directly compared the coefficient ab (indirect effect), c’ (direct effect) and c (total effect). This method is widely used and has been proved to have a higher statistical power compared with other mediating effect testing methods [64,65]. The results show that at the 95% confidence interval (CI), the ab product term does not contain zero, indicating that as a mediating variable, comfort level exerts a significant indirect effect on the relationship between a sties’ number of visitors and PM2.5 concentration. In addition, ab values and c’ values are plus-minus, meaning that both direct and indirect effects are significant, and the comfort level serves as a partial mediating variable. The total effect of PM2.5 concentrations on the number of visitors equals the direct effect –0.033 plus the indirect effect 0.015, namely –0.018, and the indirect effect accounts for 83.3% (Table 6, Figure 8).

#### 3.3.2. Correlation between Visitor Activities and Greenspace Elements

A correlation analysis of visitor activities and greenspace elements shows that physical activities have no obvious correlation with greenspace elements. For social activities however, there was a strong negative correlation with the sites’ water coverage (*R* = −0.845, *p* < 0.05). In other words, the higher proportion a site’s area is covered in water features, the lower the proportion of social activities visitors will be engaged in such as chatting and meeting friends. Overall, there is no significant linear correlation between visitor activities and greenspace elements (Table S3).

Tallying the number of visitors in the six sites showed that Site A1 was the most popular, with a green coverage rate of 37.61%, a fully open airflow of 74.5%, a semi-open airflow of 55.82%, a SVF of 0.543 and no water features. Being close to the urban artery, the least popular site was Site B, with a green coverage rate of 23.9%, a fully open airflow of 83.76%, semi-open airflow of 66.68%, and a SVF 0.65. Site B also had no water features and had high noise levels.

## 4. Discussion

### 4.1. The Greater Impact of Time on PM2.5 Concentrations Compared to Space

This paper found that more than 70% of the selected greenspaces during the observed times had PM2.5 concentrations at moderate to severe levels, and that overall air quality was poor. Among the six sites, the average PM2.5 concentrations of Sites D1 and D2, which are both adjacent to the riverfront, ranked the highest, yet due to the water-loving nature of people, these sites also attracted more visitors who tended to participate in riverside activities. This highlights the conflict between people’s outdoor preferences and behaviors with the potential health risks caused by air quality in greenspaces. From a macro perspective, the differences in directly monitored PM2.5 concentrations across the six sites ranged a limited amount from 0.53% to 2.31%. However, from a micro perspective, this study’s further analysis on the difference values shows that PM2.5 concentrations are significantly correlated with the sites’ spatial location. Although this study’s six sites are dispersed and not located in the same urban park, they all are located near Nanjing’s city center and along the same urban artery. As such, they also are all similarly affected by the influence of traffic pollution. The sites’ diverse landscape features, and the varying behavioral preferences of visitors lead to different visitor densities at the sites, while residents’ activities, such as dancing, jogging and smoking, may cause secondary particular matter. The result is a complex set of dynamic impacts on the PM concentrations among different spaces.

In terms of the temporality trends, the directly measured PM2.5 concentrations of the six sites collectively fluctuated as a whole across the survey’s 23 observed days, and this is assumed to be related to the specific weather conditions and meteorological factors affecting all the sites similarly on any given day. However, according to the temporal distributions of PM2.5 concentrations across different time periods within individual days, concentrations in the greenspaces were found to be greatly affected by road traffic pollution sources, with concentrations in the afternoon being lower than the morning presumably due to the heavier vehicle emissions of the morning rush hour. It is thus recommended that residents of the surrounding areas should avoid going out and visiting the greenspaces during the morning peak hours when PM concentrations are higher, and rather go at 13:30–16:30 when the concentrations are lower. During the morning, Site D2′s 35-m hill appears to provide a shielding effect from the traffic pollution, and thus has lower PM2.5 concentrations compared to the other sites. As an example, this suggests that outdoor morning activities should be carried out in greenspaces that are far from urban arteries, blocked by hills and with relatively large rates of water coverage. In contrast, the PM2.5 concentrations in the afternoon at Sites D1 and D2 are relatively high compared to the other sites, while Site A2 in the center of Hanzhongmen Square had the lowest. Therefore, this suggests that afternoon activities should be carried out in greenspaces in higher degrees of airflow and SVF.

### 4.2. Smog and Haze Reduction Strategies Based on Optimized Greenspace Elements

This study has found that PM2.5 concentrations of its six research sites were closely correlated with their degree of green coverage, airflow openness and meteorological factors. According to the analysis on meteorological factors in Section 3.2.2, PM2.5 concentrations had a very significant positive correlation with the sites’ humidity which, in turn, is significantly affected by the sites’ degree of green coverage and the airflow openness. Meanwhile, the analysis of greenspace elements in Section 3.2.1 showed that PM2.5 concentrations were significantly negatively correlated with airflow openness and positively correlated with green coverage. These findings are consistent with those of Yin (2007) [66] and Yang (2017) [67]. However, as described in this study’s introduction, the impacts of green coverage and plant community structure on PM2.5 concentrations remain multifaceted and can even have opposing effects depending on other variables and conditions. The multi-layered composite structures of vegetation including trees, shrubs and grass with a high canopy densities and plant coverage may have higher PM concentrations than more singular-type lawn, shrub and grass environments [68,69]. Excessive plant density and canopy density can hinder the dilution and diffusion of PMs and thereby increase their concentrations [48]. This is because high plant densities and green coverage tend to lead to higher humidity, and relatively poor airflow, impeding the diffusion of PM2.5 and other particles. Moreover, within a certain range of wind speeds, air channels provide the necessary space for air flow, allowing for the significant migration and diffusion of PM and its concentration within a site. However, this study found that there was no significant linear correlation between SVF and PM2.5 concentrations, although SVF was significantly positively correlated with the fully open airflow, thus SVF did appear to indirectly affect PM2.5 concentrations at the sites. This study also found that the sites’ water coverage has little effect on their PM2.5 concentration and humidity, conforming to existing research findings. In China, the main factors of the naturally measured water consumption of urban development land are the evaporation and transpiration of vegetation within greenspaces, known as evapotranspiration [70]. In summer and autumn, the changes in river evapotranspiration trends are very noticeable while the variations in spring and winter are basically flat [71]. Considering this and this study’s limited research period during the winter, it is expected that the influence of water coverage on the sites’ humidity is not as great as that of green coverage, and its effect on PM2.5 concentration is also correspondingly weak.

In general, the degree of the sites’ green coverage and airflow openness were the two most important factors affecting greenspace air quality, as well as the two major factors affecting the spatial pattern of the greenspace elements. Based on this, the 23 days of PM2.5 concentration data were ordered from the best to worst according to their air quality, and each site’s average PM2.5 concentration levels for corresponding air quality intervals were calculated. Then, the optimal intervals of green coverage and airflow openness were deduced from the two sites with the lowest average value (Figure S4, Table S4).

The mode of air pollution of each greenspace site was then calculated by analyzing the PM2.5 concentration data of the study period. According to the degree of green coverage and airflow openness with the best performance in the above air quality intervals, the following improvement measures are proposed for the current greenspace elements of the six sites:

1. Adjust airflow. For example, in Site A2, the degree of fully open airflow can be reduced to between 71–77% by adding small landscape buildings, while semi-open airflow can be decreased to between 45–60% by planting tall trees.
2. Adjust green coverage. For example, in Site B, the degree of green coverage can be increased to between 37% and 47% by planting more vegetation, while airflow can be maintained at its current status.
3. Jointly adjust airflow openness and green coverage. For example, in Site C, the fully open airflow can be lowered to 71–77% by adding small landscape buildings, and the semi-open airflow can be reduced to 45–60% by planting tall trees. Meanwhile, the degree of green coverage can be increased to 37–47%.

Similar suggestions can be made for the other sites based on this analysis. Furthermore, other measures can be taken at all sites to improve the thermal and humidity environments by adjusting the types of underlying surfaces that form the site and improving the spatial pattern of vegetation, so as to indirectly help reduce the PM concentrations and improve air quality.

### 4.3. Elderly Visitor’s Weak Sensitivity to Smog and Haze in Urban Greenspaces and Corresponding Potential Risks to Their Physical and Mental Heath

This study has found that elderly visitors of the research sites are not sensitive to the smog and haze in such greenspaces. Due to the decline in physical function among elderly populations, exposure to PM2.5 can cause greater and more severe harm to their physical and mental health (Text S1). In addition, towards their decisions regarding outdoor activities, this study found the elderly are relatively sensitive to changes in climate compared to smog and haze, while the comfort index appears to play an intermediary role in the process of smog and haze affecting crowd activities.

Using questionnaires and interviews, this study explored the overall perception of the elderly population towards smog and haze and their decision process towards going out to visit a greenspace. Questions included whether they had a sense of the current day’s air pollution levels and what they felt about the actual PM2.5 concentrations levels, and whether there is certain PM2.5 concentration level that they considered a special concern. Among 200 interviewees, six stated that they felt sensitive or physically unwell due to smog and haze from a recent day, and six others expressed quantitative knowledge of smog and haze. This paper focuses on the answers of these 12 respondents to explain a typical type of elderly visitor of the greenspaces. As shown in Table S5, among these 12 elderly persons, the ratio of male to female was 1:5, suggesting that women may be more sensitive to air quality and may tend to pay more attention to health problems related to smog and haze than men. For most of the set of elderly interviewees with quantitative knowledge of smog and haze, they stated their maximum acceptable value for PM2.5 concentrations when going out was 150 μg/m³, which is considered moderately polluted. A few stated they would go to the park only when the air was good quality or lightly polluted at 100 μg/m³ or less, and they may relax their standards if it is a sunny day. The other six interviewees were able to describe the exact dates on which they felt sensitive or uncomfortable due to smog and haze. The measured concentrations on these dates were all verified as being above 250 μg/m³, but they still insisted in going out to visit the greenspaces. This example shows how in their healthy pursuit of exposure to greenspaces, the elderly can also inadvertently expose themselves to unhealthy air pollution.

## 5. Conclusions

This study found that the air quality of small greenspaces dispersed within high-density central urban districts of Nanjing is non-ideal and poses a threat to human health. In terms of spatial and temporal distribution, overall, there is no significant difference in PM2.5 concentrations between different greenspaces. However, when it comes to the difference values, the distance from a shared urban artery has a notable influence on air quality between the different sites. In addition, the PM2.5 concentrations of different greenspaces show significant variations in their temporal distributions. Due to the traffic pollution caused mainly by vehicle emissions during the morning rush hour period, the PM2.5 concentrations in the morning are higher than in the afternoon. In terms of physical factors, greenspace elements such as green coverage and airflow openness show remarkably positive and negative correlations with PM2.5 concentrations respectively. SVF presents a significantly positive correlation with the degree of fully open airflow, which indirectly affects the PM2.5 concentration at the sites. Meteorological factors such as temperature and humidity are very significantly positively correlated with PM2.5 concentrations, while comfort level has a significantly negative correlation. Furthermore, humidity is also significantly influenced by green coverage and airflow openness. Based on the above findings, the optimal green coverage and airflow openness for different air quality intervals were calculated and were used to formulate optimized smog and haze reduction strategies. According to the mode of air quality in the six greenspaces during the study period, corresponding improvement measures were then proposed based on the aforementioned strategies.

The elderly are the main users of greenspaces yet this study’s questionnaire and interview findings showed they tend to be insensitive to smog and haze when deciding whether to visit a greenspace for physical and social activities. Instead, they are more likely to be relatively sensitive to changes in the local climate. The comfort level exerts a notable mediating effect in the process of PM2.5 concentration affecting greenspace visitor activities. Currently, the well-being of the elderly has drawn global attention and this study provides relevant insights regarding the spatial and temporal distribution of PM2.5 concentrations in China’s greenspaces where the surrounding elderly communities tend to carry out many of their daily activities. With these insights, this paper intends to spark discussion for improving greenspace quality and overall livable conditions of high-density central urban districts in the hope of providing a theoretical support and reference for elderly-oriented greenspace construction and indicators in the future.

## Figures and Tables

**Table 1:** Research site data and observed activities.

Greenspace Code	Name of Greenspace		Location Characteristics		Distance from Urban Artery
A	Hanzhongmen City Square		Adjacent to street		125 m
	Site Code	Description	Activity Types	Proportion	Observed Activities
	A1	Shady greenway on north side of the square, 86 m away from urban artery	social	97.5%	Playing chess and cards, chatting and sitting idly, etc.
			physical	2.7%	Speed walking, exercising, etc.
	A2	Center of Hanzhongmen Square, 125 m away from urban artery	social	82.4%	Dancing, taking children on walks, etc.
			physical	39.2%	Playing ball games, speed walking, etc.
B	Zhengdayuan Small-scale Greenspace		Adjacent to street and riverfront		46 m
	B	Equipped with fitness facilities	social	82.4%	Dancing, taking children on walks, etc.
			physical	39.2%	Playing ball games, speed walking, etc.
C	Qinhuai Riverfront Greenspace		Riverfront		118 m
	C	An open platform in the middle, 118 m away from urban artery	social	21.1%	Chatting and sitting idly, etc.
			physical	83.1%	Speed walking, running, walking the dog, etc.
D	Stone City Park		Riverfront		330 m
	D1	Stone City Square, 316 m away from urban artery	social	86.7%	Singing, dancing, playing instruments, etc.
			physical	77.8%	Visiting, photographing, etc.
	D2	“Ghost Face” scenic spot between the hill and river, 460 m away from urban artery	Social	75.5%	Chatting, playing instruments, flying kites, etc.
			Physical	60.7%	Visiting, jogging, etc.

**Table 2:** PM2.5 concentrations across research sites.

Sites	A1	A2	B	C	D1	D2
Average PM2.5 Concentration (μg/m³)	187.5	185.0	188.9	185.6	192.7	190.3
Optimal (≤35 μg/m³) * & Good (35 < PM2.5 ≤ 75 μg/m³) * Percentage	8.7%	13.0%	8.7%	13.0%	13.0%	13.0%
Lightly Polluted (75 < PM2.5 ≤ 115 μg/m³) * Percentage	17.4%	13.0%	17.4%	13.0%	8.7%	8.7%
Moderately Polluted and above (>115 μg/m³) * Percentage	73.9%	74.0%	73.9%	74.0%	78.3%	78.3%

* For the specific indicators, please refer to Text S1.

**Table 3:** Physical factor index of research sites.

Site	Factor Indicator		Schematic Diagram	SVF
A1	Green Coverage	37.61%	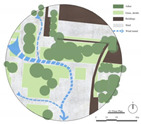	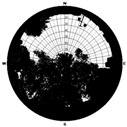
	Water Coverage	0.00%		
	Degree of Fully Open Airflow	74.50%		
	Degree of Semi-open Airflow	55.82%		
	SVF (sky view factor)	0.543		
A2	Green Coverage	47.21%	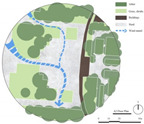	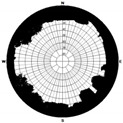
	Water Coverage	0.00%		
	Degree of Fully Open Airflow	94.72%		
	Degree of Semi-open Airflow	68.42%		
	SVF	0.752		
B	Green Coverage	23.90%	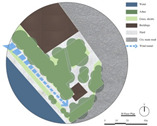	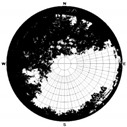
	Water Coverage	11.17%		
	Degree of Fully Open Airflow	83.76%		
	Degree of Semi-open Airflow	66.68%		
	SVF	0.650		
C	Green Coverage	15.71%	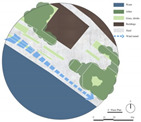	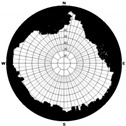
	Water Coverage	31.74%		
	Degree of Fully Open Airflow	90.22%		
	Degree of Semi-open Airflow	78.01%		
	SVF	0.757		
D1	Green Coverage	63.71%	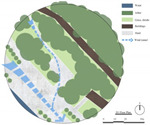	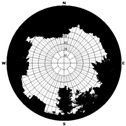
	Water Coverage	1.02%		
	Degree of Fully Open Airflow	71.98%		
	Degree of Semi-open Airflow	47.83%		
	SVF	0.610		
D2	Green Coverage	55.29%	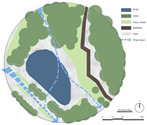	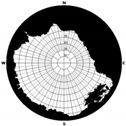
	Water Coverage	13.31%		
	Degree of Fully Open Airflow	77.18%		
	Degree of Semi-open Airflow	54.93%		
	SVF	0.700		

**Table 4:** Correlation of PM2.5 concentration and greenspace elements.

	Correlation Analysis		Green Coverage	Water Coverage	Semi-Open Airflow	Fully Open Airflow	SVF
Total	PM2.5	R	0.628	0.691	−0.830 *	−0.838 *	−0.506
		S	0.182	0.129	0.041	0.037	0.306
Sunny, Cloudy Days, Wind Speed > 0.3 m/s	PM2.5	R	0.819 *	−0.720	−0.887 *	−0.689	−0.397
		S	0.046	0.170	0.018	0.130	0.436
Sunny, Cloudy Days, Wind Speed > 1.5 m/s	PM2.5	R	0.786	0.508	−0.833 *	−0.601	−0.520
		S	0.064	0.304	0.040	0.208	0.290

Note: * *p* < 0.05.

**Table 5:** Correlation table of PM2.5 concentration and meteorological factors.

Pearson Correlation Analysis		Temperature	Humidity	Wind Speed	Wind Direction	Comfort Level
PM2.5	R	0.174 *	0.541 **	−0.103	0.037	−0.400 **
	S	0.041	0.000	0.230	0.668	0.000

Note: ** *p* < 0.01; * *p* < 0.05.

**Table 6:** Mediating effect testing results.

Item	c-Total Effect	a	b	a × b Indirect Effect	a × b (95% CI)	c’-Direct Effect	Conclusion	Effect Proportion Formula	Effect Proportion
PM2.5 ≥ Comfort Level ≥ Number of Visitors	−0.018	−0.008 **	−1.875 *	0.015	0.019~0.157	−0.033 *	partial mediating variable	|a × b/c|	83.3%

Note: ** *p* < 0.01; * *p* < 0.05.

## Data Availability

The data presented in this study are available on request from the corresponding author.

## Supplementary Materials

The following are available online at https://www.mdpi.com/article/10.3390/ijerph18189705/s1, Figure S1: Diagram of Overall Research Process, Figure S2: Site Activity Photos, Figure S3: Comparison of PM2.5 concentrations across research sites, Figure S4: Directly measured PM2.5 concentrations by level, Table S1: Correlation of greenspace elements, Table S2: Correlation table of visitor activities, meteorological factors and PM2.5 concentrations, Table S3: Correlation table of visitor activities and greenspace elements, Table S4: Optimal and worst intervals of greenspace elements for each air quality interval, Table S5: Interview records of elderly people regarding awareness of smog and haze, Text S1: The Technical Regulation on Ambient Air Quality Index (HJ633-2012).
