# Supplementary material for: PM2.5 Pollutant Concentrations in Greenspaces of Nanjing Are High but Can Be Lowered with Environmental Planning
Source: Int J Environ Res Public Health. 2021 Sep 15;18(18):9705. doi: 10.3390/ijerph18189705 (PMC8470726; doi:10.3390/ijerph18189705)
Supplement: Supplementary file 1 [file ijerph-18-09705-s001.zip › ijerph-1370949-supplementary.pdf]

Supplementary Materials

Text S1. The Technical Regulation on Ambient Air Quality Index (HJ633-2012)

According to the Technical Regulation on Ambient Air Quality Index (HJ633-2012) issued by China's Ministry of Ecology and Environment (2012) [57], when the average PM2.5 concentration is less than or equal to 35μg/m³, the air quality is classified as optimal, when it is 35 < PM2.5 ≤ 75μg/m³, the air quality is classified as good; when it is 75 < PM2.5 ≤ 115μg/m³, lightly polluted; 115 < PM2.5 ≤ 150μg/m³, moderately polluted, children and the elderly should avoid long time, high intensity outdoor exercise, the general population should reduce outdoor exercise; 150 < PM2.5 ≤ 250μg/m³, heavily polluted, children and the elderly should stay indoors, stop outdoor exercise, the general population should reduce outdoor exercise; and PM2.5 > 250μg/m³, severely polluted, children and the elderly should stay indoors to avoid physical consumption, and the general population should avoid outdoor activities.

References

57. MOEE. Technical Regulation on Ambient Air Quality Index.; 2012. http://www.mee.gov.cn/ywgz/fgbz/bz/bzwb/jcffbz/201203/t20120302_224166.shtml

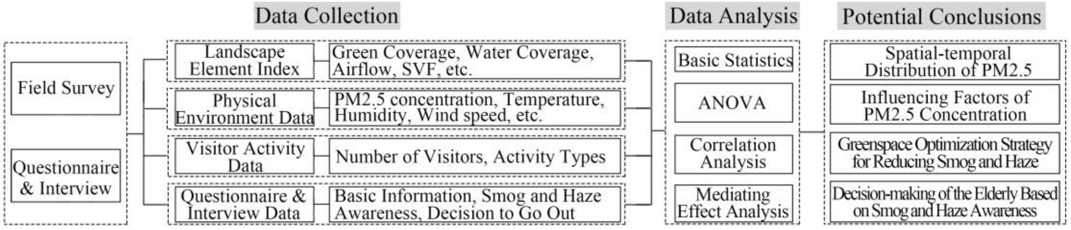

Figure S1. Diagram of Overall Research Process

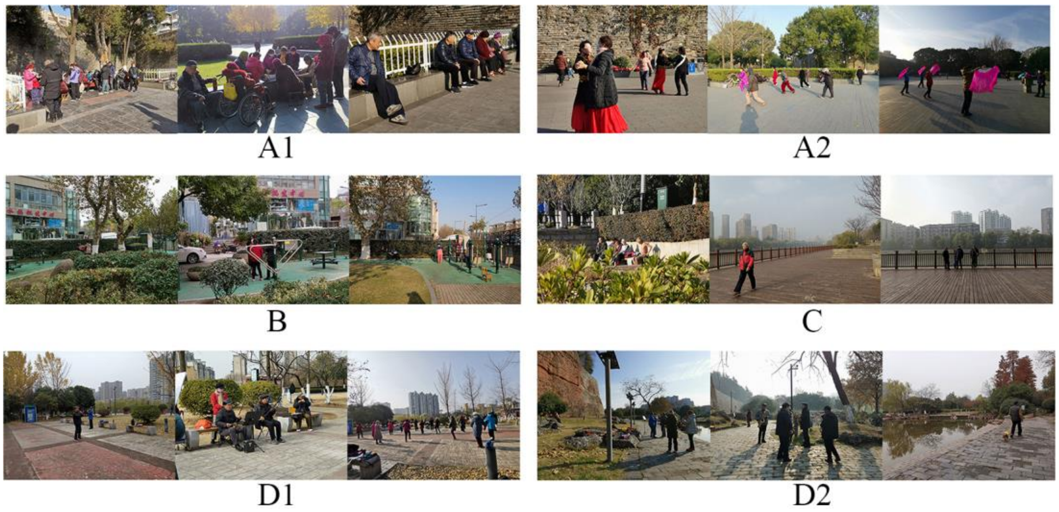

Figure S2. Site Activity Photos

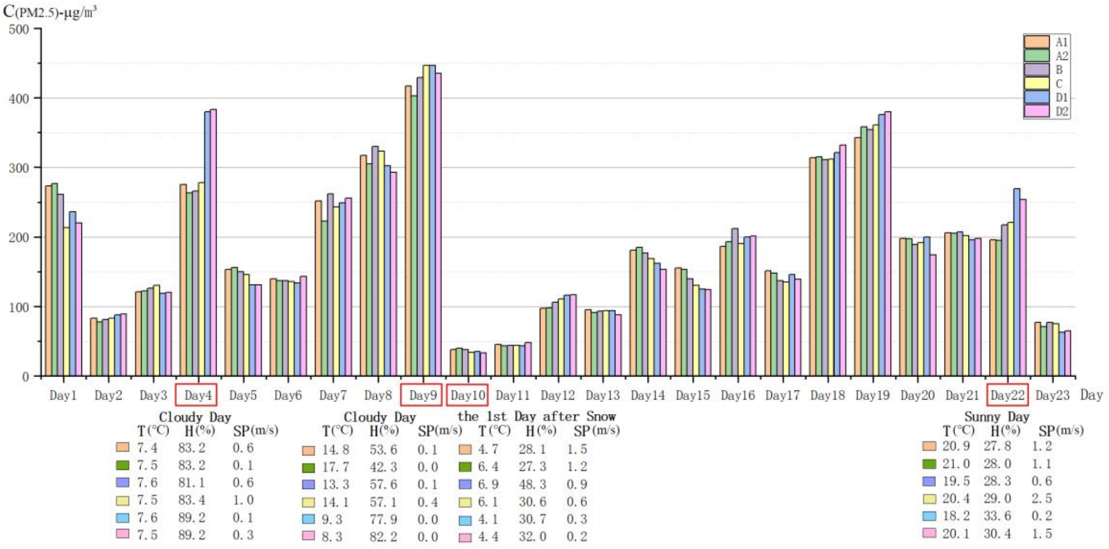

Figure S3. Comparison of PM2.5 Concentrations Across Research Sites

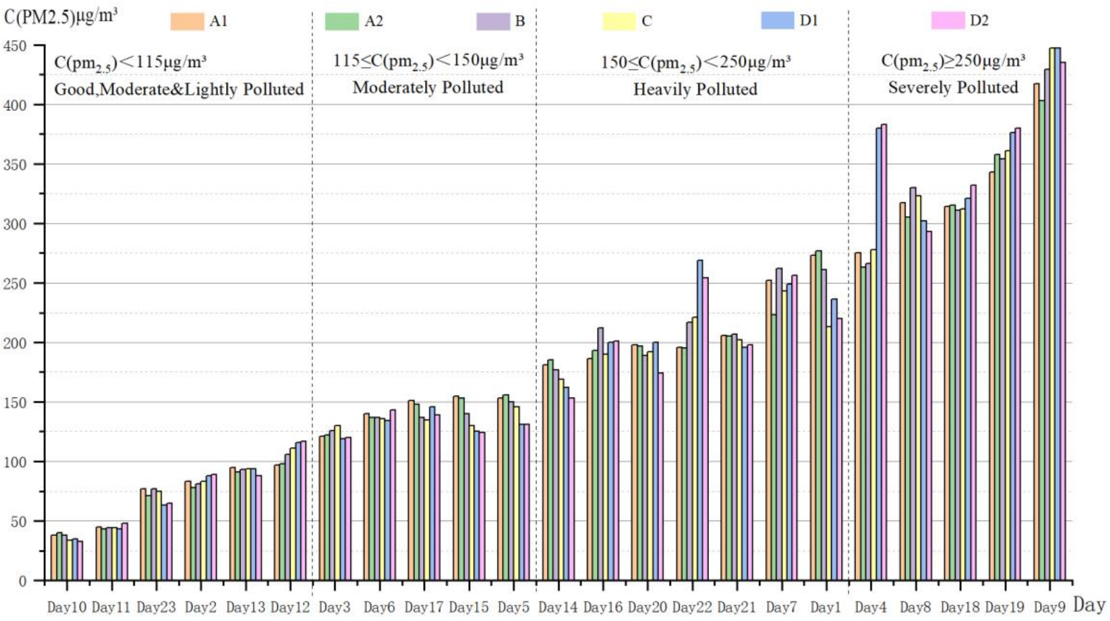

Figure S4. Directly Measured PM2.5 Concentrations by Level

Table S1. Correlation of Greenspace Elements.

Correlation Analysis		Green Coverage (a)	Water Coverage (b)	Semi-Open Airflow (c)	Fully Open Airflow (d)	SVF（e）
（a）	R	1	-0.663	-0.833*	-0.513	-0.239
	S		0.151	0.040	0.298	0.648
（b）	R		1	0.667	0.361	0.568
	S			0.148	0.482	0.239
（c）	R			1	0.886*	0.701
	S				0.019	0.121
（d）	R				1	0.821*

S	.045

Note: * $p < 0.05$

Table S2. Correlation Table of Visitor Activities, Meteorological Factors and PM2.5 Concentrations

Pearson Correlation		Comfort Level	Temperature	Wind Speed	Humidity	PM2.5
Number of Visitors	R	-0.131	0.223**	0.017	-0.252**	-0.104
	S	0.125	0.008	0.840	0.003	0.226
Proportion of Social Activities	R	-0.074	0.057	-0.077	-0.022	0.012
	S	0.390	0.504	0.367	0.802	0.889
Proportion of Physical Activities	R	0.030	-0.056	-0.039	0.033	0.079
	S	0.724	0.517	0.648	0.697	0.355

Note: ** $p < 0.01$; * $p < 0.05$

Table S3. Correlation Table of Visitor Activities and Greenspace Elements

Correlation Analysis		Green Coverage	Water Coverage	Fully Open Airflow	Semi-Open Airflow	SVF
Number of Visitors	R	0.057	-0.252	0.180	0.075	-0.037
	S	0.914	0.630	0.734	0.888	0.944
Proportion of Social Activities	R	0.799	-0.845*	-0.495	-0.763	-0.514
	S	0.057	0.034	0.318	0.077	0.297
Proportion of Physical Activities	R	-0.193	0.573	0.150	0.257	0.436
	S	0.715	0.235	0.776	0.623	0.387

Note: * $p < 0.05$

Table S4. Optimal and Worst Intervals of Greenspace Elements for Each Air Quality Interval

Air Quality Interval	O-Green Coverage	W- Green Coverage	O-Fully Open Airflow	W-Fully Open Airflow	O-Semi-Open Airflow	W-Semi-Open Airflow
PM2.5＜115μg/m³ Optimal, Good, Lightly Polluted	15%-24%	55%-64%	90%-99%	80%-89%	60%-69%	45%-60%
115μg/m³≤PM2.5＜150μg/m³, Moderately Polluted	55%-64%	37%-47%	71%-77%	90%-99%	45%-60%	60%-79%
150μg/m³≤PM2.5＜250μg/m³, Heavily Polluted	37%-47%	15%-24%	71%-77%	80%-89%	45%-60%	60%-69%
PM2.5≥250μg/m³, Severely Polluted	37%-47%	55%-64%	80%-89%	71%-77%	60%-69%	45%-60%

Table S5. Interview Records of Elderly People Regarding Awareness of Smog and Haze.

No.	Gender	Age	Health Condition	Acceptable Threshold or Sensitive Value of PM2.5 Concentration	Notes
I. People with Quantitative Cognition of Smog and Haze					
1	M	50-60	Good	≤150μg/m³	Familiar with PM2.5 standards in Taiwan, Mainland China and international community. Very concerned about smog and haze, and uses his own methods for identifying and

					monitoring excessive PM. When the concentration of PM2.5 exceeds 100μg/m³, he will wear a mask.
2	M	70-79	Indigestion	≤100μg/m³	Aware of the PM2.5 index; avoids going out when the PM2.5 concentration exceeds 100μg/m³, unless it's a sunny day.
3	F	70-79	Good	≤140μg/m³	Aware of the PM2.5 index, will consider not going out when the PM2.5 concentration exceeds 140μg/m³, unless it's a sunny day. Checks the PM2.5 index when it's a hazy weather.
4	F	60-69	Good	≤100μg/m³	Aware of the PM2.5 index and avoids going out when the PM2.5 concentration exceeds 100μg/m³. Believes compared with other cities such as Fuzhou and Haikou, Nanjing's air quality is worrying.
5	F	50-59	Good	≤150μg/m³	Has an air quality monitor at home and avoids going out when the monitor reports an indoor PM2.5 concentration of 75. But if it's a sunny day, she may still go out.
6	F	50-59	Good	≤150μg/m³	Aware of the PM2.5 index and will go out when the air quality is optimal or good, avoid going out when lightly polluted.
II. People who have an awareness of smog and haze on the current day but still chose to go out					
7	F	70-79	Good	Measured Value: 273μg/m³	Sensitive to the air in Hanzhongmen Square in the afternoon of December 3, 2020
8	F	60-69	Good	Measured Value: 250μg/m³	Sensitive to the air in Stone City Park in the morning of December 10, 2020
9	F	60-69	Poor Respiratory System	Measured Value: 252μg/m³	Sensitive to the air in Hanzhongmen Square in the morning of December 10, 2020
10	F	70-79	Good	Measured Value: 300μg/m³	Sensitive to the air in Stone City Park in the morning of December 11, 2020
11	F	70-79	Good	Measured Value: 320μg/m³	Sensitive to the air in Stone City Park in the afternoon of December 23, 2020
12	F	60-69	Good	Measured Value: 320μg/m³	Sensitive to the air in Stone City Park in the afternoon (cloudy) of December 23, 2020; Not Sensitive to the air in the afternoon (Sunny, PM2.5 reached 370μg/m³) of December 24, 2020, due to a late going-out time of 10 am when the smog and haze had dispersed
